# A comparison of the functional parameters of operability in patients with post-inflammatory lung disease and those with lung cancer requiring lung resection

**DOI:** 10.7196/AJTCCM2018.v24i1.158

**Published:** 2018-04-03

**Authors:** M H Amirali, E M Irusen, C F N Koegelenberg

**Affiliations:** Division of Pulmonology, Department of Medicine, Stellenbosch University and Tygerberg Academic Hospital, Cape Town, South Africa

**Keywords:** lung cancer, lung resection, operability, South Africa

## Abstract

**Background:**

It is a common, yet unproven, belief that patients with post-inflammatory lung disease have a better functional reserve than patients with lung cancer when compared with their respective functional parameters of operability – forced expiratory volume in one second (FEV_1_), maximum oxygen uptake in litres per minute (VO_2_ max) and the diffusion capacity for carbon monoxide (DLCO).

**Objectives:**

The aim of this study was to compare a group of patients with lung cancer with a group with post-inflammatory lung disease according to their respective functional parameters of operability. We also aimed to investigate any associations of FEV_1_ and/or DLCO with VO_2_ max within the two groups.

**Methods:**

We retrospectively included 100 adult patients considered for lung resection. All patients were worked up using a validated algorithm and were then sub-analysed according to their parameters of functional operability.

**Results:**

Two-thirds of patients had post-inflammatory lung diseases whilst the rest had lung cancer. The majority of the patients in the lung cancer group had coexistent chronic obstructive pulmonary disease (COPD) (*n*=18). Most (*n*=47) of the patients in the post-inflammatory group were diagnosed with a form of pulmonary TB (active or previous). Among the two groups, the lung cancer group had a higher median %FEV_1_ value (62.0%; interquartile range (IQR) 51.0 - 76.0) compared with the post-inflammatory group (52%; IQR 42.0 - 63.0; *p*=0.01). There was no difference for the %DLCO and %VO_2_ max values. The lung cancer group also had higher predicted postoperative (ppo) values for %FEV_1_ (41.0%; IQR 31.0 - 58.0 v. 34.0%; IQR 23.0 - 46.0; *p*=0.03, respectively) and %VO_2_ max (58.0%; IQR 44.0 - 68.0 v. 46.0%; IQR 35.0 - 60.0; *p*=0.02). There was no difference in the %DLCO ppo values between the groups.

**Conclusion:**

Patients with lung cancer had higher percentage values for FEV_1_ and ppo parameters for %FEV_1_ and %VO_2_ max compared with those who had post-inflammatory lung disease. Our findings suggest that lung cancer patients have a better functional reserve.

## Background

Cancer is one of the leading causes of mortality worldwide. Lung cancer is the leading cause of cancer-related mortality globally, causing 1.6 million deaths in 2012.^[1]^ However, in southern Africa, the relationship between lung cancer and its mortality rate remains low in comparison with other cancers and respiratory diseases.^[2–5]^

According to the World Health Organization (WHO), an estimated 7.7 million cases of pulmonary tuberculosis (PTB) occurred worldwide in 2007^[6]^ and South Africa (SA) had the third highest tuberculosis (TB) burden.^[7,8]^ Treated PTB can lead to complications, including progressive loss of lung function, persistent pulmonary symptoms^[9]^ and chronic pulmonary aspergillosis.^[10–12]^ These complications frequently necessitate surgery. A study by Rizzi *et al*. ^[13]^ reported that patients with post tuberculous chronic haemoptysis (10.0%), lung destruction (8.1%), chest wall involvement (1.9%), suspected cancer (24.2%), cavitatory lung disease (21.9%) and bronchiectasis (16.1%) required elective surgery, whereas those with massive bleeding (5.4%) or a bronchopleural fistula (3.1%) required emergency surgery.

Lung resection can be a high-risk procedure, especially in patients with underlying cardiopulmonary disease. Predictors of mortality include the extent of resection, comorbidities and cardiopulmonary reserve.^[14,15]^

Ninety percent of lung cancer patients are current or past smokers, which is frequently associated with varying degrees of concomitant chronic obstructive pulmonary disease and/or ischaemic heart disease. Furthermore, many of these patients are of advanced age and this places them at an increased risk of post-operative complications and mortality.^[16,17]^ A number of prospective studies have validated a percentage-predicted forced expiratory volume in one second predicted postoperative value (%FEV_1_ ppo) of <40% as a prohibitive threshold for pulmonary resection, with mortality rates as high as 50% in such patients. Ferguson *et al*. ^[18]^ demonstrated that a diffusion capacity for carbon monoxide (DLCO) of <60% of the predicted value was a cut-off value for major pulmonary resection. The maximum oxygen uptake in litres per minute predicted postoperative (VO_2_ max ppo) value of <10 ml/kg/min, obtained from either formal cardiopulmonary exercise testing (CPET) or low-technology (minimal achievement) exercise tests, is associated with a high risk of post-operative complications and death. Regarding the cardiac risk assessment, the Revised Cardiac Risk Index (RCRI)^[19]^ is used by many authorities. The criteria contain six independent variables that correlate with post-operative cardiac complications - these include a high-risk type of surgery, a history of ischaemic heart disease, cardiac failure, cerebrovascular disease, diabetes requiring treatment with insulin and pre-operative serum creatinine of >177 µmol/L. Patients with more than two variables have a postoperative cardiac complication rate >10% and are considered to be at high risk.^[17]^

The validated algorithms used to assess candidates for lung resection are based on spirometry, the DLCO and the VO_2_ max.^[14]^ One such algorithm proposed by Bolliger and Perruchoud^[15]^ has been used widely as a tool for evaluating cardiorespiratory reserves of lung resection candidates. The algorithm proposes that patients undergo successive steps of functional testing, the results of which qualify them for varying extents of resection or alternatively preclude them from any surgery.^[15]^

**Fig. 1:**
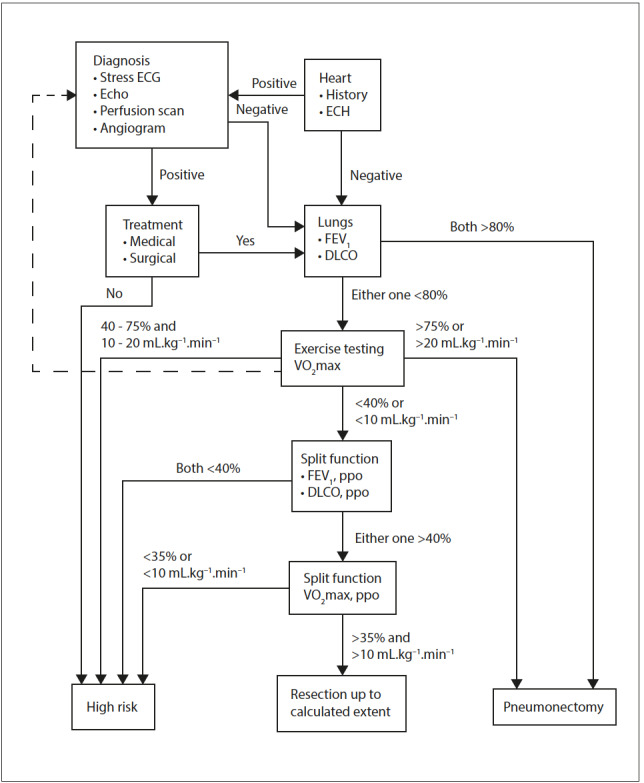
Algorithm proposed by Bolliger *et al*.,^[15]^ adapted by Koegelenberg *et al*.^[17]^ ECG = electrocardiogram FEV_1_ = forced expiratory volume in one second DLCO = diffusion capacity for carbon monoxide VO_2_ max = maximum oxygen uptake in litres per minute mL = millilitres kg = kilograms

Apart from the underlying cardiopulmonary disease and other comorbidities, the calculated predicted postoperative (ppo) values for FEV_1_ , VO_2_ max and DLCO are directly proportional to postoperative functional state and mortality.^[21]^

It is a commonly held belief by various experts in the field of pulmonology that patients with post-inflammatory lung disease have a better functional reserve postoperatively than patients with lung cancer, when comparing their respective FEV_1_ , VO_2_ max and DLCO values; however, there is limited evidence to support the belief.^[16]^

The aim of the present study was to compare two groups of patients (i.e. patients with lung cancer v. patients with post-inflammatory lung disease), and to investigate the association of functional parameters of operability within these two groups of patients.

## Methods

### Study design and population

We retrospectively enrolled adult patients who had been considered for lung resection and were referred to the Division of Pulmonology at Tygerberg Academic Hospital, Cape Town, with either lung cancer or post-inflammatory lung disease. Ethical approval for this retrospective analysis was obtained from the Stellenbosch University Research Ethics Committee (ref. no. S15/04/074). The application included a waiver of consent due to the retrospective nature and anonymity of the study design.

**Fig. 2:**
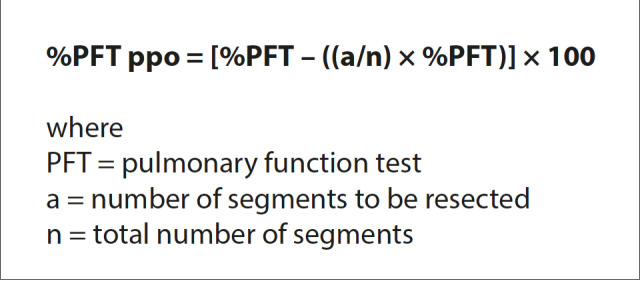
Equation used to calculate %PFT ppo value. ppo = predicted postoperative PFT = pulmonary function test

Cases were identified from existing medical records; they were stratified into two groups, namely ‘A’ and ‘B’, where ‘A’ comprised patients with non-small-cell lung cancer while ‘B’ comprised patients with post-inflammatory lung disease (bronchiectasis, active/post tuberculous haemoptysis, and aspergilloma). After obtaining permission from the chief medical superintendent, the original medical records of all cases identified were requested and data were collected anonymously. The data collected included the demographics (age, gender), comorbidities of patients, indications for lung resection, extent of lung resection, and their pulmonary function test values (i.e. FEV_1_ , FVC, DLCO and VO_2_ max). The ppo value for these parameters can be calculated by the equation in Fig. 2, where the pulmonary function test (PFT) can either be %FEV_1_ , %VO_2_ max or %DLCO. We used three validated ways of estimating the relative functional contribution or split function, i.e. anatomical calculation, split radionucleotide perfusion scanning and quantitative computer tomography scanning and dynamic perfusion magnetic resonance imaging (MRI).

Anatomical calculations of ppo values were performed on all patients who required pre-operative estimation of post-operative lung function. Patients who required further evaluation underwent either radionucleotide perfusion scanning or quantitative CT scanning. All patients were worked up for lung resection using the algorithm for the assessment of their cardiorespiratory reserves (functional operability).^[17]^ Patients were generally followed up as outpatients and CPET was only performed once the risk of haemoptysis was evaluated (i. e. no haemoptysis for 2 weeks). Patients included in the study were then evaluated for their respective functional operability parameters.

### Statistical analysis

χ2 comparisons and Pearson product-moment correlation coefficient (Pearson’s *r* or ‘*r*-squared’) of proportional data were performed. We did not make any assumptions for normality; hence, these nonparametric inferences were used for statistical analysis. A *p*-value <0.05 in a two-tailed test of proportions (χ2 ) was considered statistically significant. Unless stated otherwise, data are displayed as median with interquartile range (IQR) values.

## Results

We included 100 patients in our study. The demographic data, primary diagnoses and comorbidities of the patients are summarised in Table 1.The majority of our patients were male (n=66/100); 51 were diagnosed with a post-inflammatory lung disease, while the rest had lung cancer.

**Table 1:** Demographic and clinical data of study population (*N*=100)

	*n *(%)*
**Male**	66 (66.0)
**Female**	34 (34.0)
**Age (years), mean (range)**	46.7 (17 - 72)
**Medical condition**	
Lung cancer	
Male	15 (62.5)
Female	9 (37.5)
Comorbidities	
Hypertension	8 (19.0)
HIV	0 (0.0)
Pulmonary TB	1 (2.4)
COPD	18 (42.9)
Smoking	11 (26.2)
CAD	2 (4.8)
None	2 (4.8)
Post-inflammatory	
Male	51 (67.1)
Female	25 (32.9)
**Diagnoses**	
Post-TB bronchiectasis	14 (19.7)
Bronchiectasis	18 (25.3)
Aspergillomata	18 (25.3)
Destroyed lung	14 (19.7)
Echinococcal cysts	3 (4.2)
Empyema	1 (1.4)
Adenomatoid malformation	1 (1.4)
Post-TB upper-lobe changes	1 (1.4)
MDR-TB	1 (1.4)
Comorbidities	
Hypertension	6 (4.30)
HIV	12 (8.70)
Pulmonary TB (active and previous)	47 (34.0)
COPD	30 (21.7)
Smoking	23 (16.7)
CAD	2 (1.4)
Bronchiectasis	1 (0.7)
None	17 (12.3)

TB = tuberculosis

COPD = chronic obstructive pulmonary disease

CAD = coronary artery disease

MDR-TB = multidrug-resistant tuberculosis

*Unless otherwise specified

The most common diagnosis in the post-inflammatory group was that of haemoptysis (*n*=47). Bronchiectasis and aspergilloma were the second most common diagnoses, followed by post-TB bronchiectasis and destroyed lung.

The majority of the patients in the lung cancer group had COPD (*n*=18), 11 of them were either active or previous smokers. Two of the patients had ischaemic heart disease. Most (*n*=47) of the patients in the post inflammatory group were diagnosed with some form of pulmonary TB (active or previous). COPD and smoking had the second and third highest prevalence, and 17 patients had no associated comorbidities.

When comparing the various functional parameters of operability between the two groups, the lung cancer group had higher %FEV_1_ values (62.0%; IQR 51.0 - 76.0; *p*=0.01), there were no differences between the %DLCO (56.0%; IQR 44.0 - 75.0; *p*=0.509), and %VO_2_ max values (80.0%; IQR 66.0 - 89.0; *p*=0.105). The lung cancer group also had higher ppo values for %FEV_1_ (41.0%; IQR 31.0 - 58.0; *p*=0.03), and %VO_2_ max (58.0%; IQR 44.0 - 68.0; *p*=0.02); there was ,however, no difference for %DLCO ppo values 40.0% (IQR 23.0 - 51.0; *p*=0.849). The values for the post-inflammatory group were: %FEV_1_ 52.0% (IQR 42.0 - 63.0); %DLCO 63.0% (IQR 51.0 - 75.0); and %VO_2_ max 72.0% (IQR 59.0 - 82.0). The ppo values were: %FEV_1_ 34.0% (IQR 23.0 - 46.0); %VO_2_ max 46.0% (IQR 35.0 - 60.0); and %DLCO 39.0% (IQR 26.0 - 55.0). Correlation analysis did not show any correlation between the two groups.

**Table 2:** Comparison of functional parameters of operability among the two groups

****	**All, median (IQR)**	A,^*^ median (IQR)	**B,^†^ median (IQR)**	***p*-value**
%FEV_1_	55 (43 - 65)	62 (51 - 76)	52 (42 - 63)	0.01
%FEV_1_ ppo	35 (26 - 48)	41 (31 - 58)	34 (23 - 46)	0.03
%VO_2_ max	73 (60 - 84)	80 (66 - 89)	72 (59 - 82)	0.105
%VO_2_ max ppo	49 (38 - 63)	58 (44 - 68)	46 (35 - 60)	0.02
%DLCO	62 (50 - 75)	56 (44 - 75)	63 (51 - 75)	0.509
%DLCO ppo	40 (26 - 54)	40 (23 - 51)	39 (26 - 55)	0.849

IQR = interquartile range

%FEV_1_ = percentage predicted for forced expiratory volume in one second

%FEV_1_ ppo = percentage predicted for forced expiratory volume in one second predicted postoperative

%VO_2_ max = percentage predicted for maximum oxygen uptake in litres per minute

%VO_2_ max ppo = percentage predicted for maximum oxygen uptake in litres per minute predicted postoperative

%DLCO = percentage predicted for diffusion capacity for carbon monoxide

%DLCO ppo = percentage predicted for diffusion capacity for carbon monoxide predicted postoperative

^*^Non-small-cell lung cancer group

^†^Post-inflammatory group (bronchiectasis, post tuberculous haemoptysis, aspergilloma)

## Discussion

We found statistically significant differences between the two groups when comparing the %FEV_1_ , %FEV_1_ ppo, and %VO_2_ max ppo; the lung cancer group had a higher %FEV_1_ (*p*=0.01), and higher ppo values for %FEV_1_ and %VO_2_ max (*p*=0.03 and *p*=0.02, respectively). We found no statistically significant differences between the two groups when we compared the %DLCO, %DLCO ppo and %VO_2_ max. No genderbased differences were observed. There was no correlation between the variables in either group. Therefore, both FEV_1_ and DLCO did not predict VO_2_ max in either group.

It is well-known that the pre-operative assessment predicts postoperative functional reserve, morbidity and mortality. Usually, a FEV_1_ ppo, DLCO ppo, and VO_2_ max ppo <40% of normal values have all been found to indicate increased mortality.^[22]^ We have shown that patients with lung cancer have a better functional reserve when compared with those who have post-inflammatory lung disease, and that neither FEV_1_ nor DLCO predicted VO_2_ max in either group. There was also no predilection of the functional reserve towards the sex or age of our patients. We believe that these findings will have implications for the surgical management of patients with lung cancer, in that they may now be more readily considered for lung resection.

Depending on the extent and the time elapsed from the operation, lung resections determine a variable reduction in functional reserve. A study by Brunelli *et al*. ^[23]^ showed that at one month after lobectomy, the FEV_1_ , DLCO, and VO_2_ max values were 79.5%, 81.5%, and 96% of preoperative values, respectively. These recovered to 84%, 88.5% and 97%, respectively, after 3 months. Regarding pneumonectomy, the %FEV_1_ , %DLCO, and VO_2_ max values were 65%, 75%, and 87% of preoperative values at 1 month, respectively; at 3 months postoperatively, the values were 66%, 80%, and 89%, respectively. Other studies have shown similar results.^[24–26]^

Inferring from these data, the lung cancer group in our study would most likely have a better overall functional reserve postoperatively. Therefore, the assumption that lung cancer patients have a worse functional reserve postoperatively when compared with patients who have post-inflammatory lung disease is untrue.

### Study strengths and limitations

This was a single-centre study, which benefits from strict adherence to a validated algorithm. The retrospective nature of the study, as well as the potential selection bias, could be limiting as only patients who were deemed clinically fit were recruited as study participants. We did not collect data on postoperative complications and mortality.

## Conclusion

We found that patients with lung cancer had higher percentage-predicted values for FEV_1_ and predicted postoperative values for %FEV_1_ and %VO_2_ compared with those who had post-inflammatory lung disease. Future prospective studies should preferably include the postoperative outcomes among the two groups to provide a comprehensive analysis.
